# Soybean glycinin improves HDL-C and suppresses the effects of rosuvastatin on hypercholesterolemic rats

**DOI:** 10.1186/1476-511X-10-165

**Published:** 2011-09-21

**Authors:** Priscila G Fassini, Roberto W Noda, Ederlan S Ferreira, Maraiza A Silva, Valdir A Neves, Aureluce Demonte

**Affiliations:** 1 Department of Food and Nutrition. Faculty of Pharmaceutical Sciences, São Paulo State University - UNESP, Araraquara, SP, Brazil; 2 Center of Applied Biology, Embrapa, Sete Lagoas, MG, Brazil

**Keywords:** cholesterol, soybean glycinin, rosuvastatin, experimental model, hypercholesterolemia

## Abstract

**Background:**

This study was an investigation of the effects of ingesting a daily dose of isolated glycinin soy protein (11S globulin), in association with rosuvastatin, on the control of hypercholesterolemia in experimental animals.

**Methods:**

Male Wistar rats were kept in individual cages under appropriate controlled conditions of temperature, light and humidity. The animals were divided into five groups (n = 9): 1) standard (STD): fed on casein as protein source; 2) hypercholesterolemic (HC): STD plus 1% cholesterol and 0.5% cholic acid; 3) HC+11S: hypercholesterolemic + glycinin (300 mg/kg/day); 4) HC+ROS: hypercholesterolemic + rosuvastatin (10 mg/kg/day); 5) HC+11S+ROS: HC diet, the 11S protein and the drug in the doses given in (3) and (4). The protein and the drug were administered by gavage for 28 days. The results indicated that the addition of 1% cholesterol and 0.5% cholic acid induced hypercholesterolemia in the animals without interfering with their weight gain.

**Results:**

A single daily dose of glycinin contributed an additional 2.8% of dietary protein intake and demonstrated its functional role, particularly in raising HDL-C, decreasing triglycerides in the liver and improving the atherogenic index in animals exposed to a hypercholesterolemic diet.

**Conclusion:**

Most of the beneficial effects of the isolated treatments disappeared when the drug (rosuvastatin) and the protein (glycinin) were taken simultaneously. The association was shown not to interact additively, as noted in the plasma levels of total cholesterol and non-HDL cholesterol, and in the significant increase of cholesterol in the liver. Studies are in progress to identify the effects of peptides derived from the 11S globulin and their role in cholesterol metabolism.

## Background

The nutritional properties of soybean proteins are well known. They have also been studied in animal models and in humans as a form of intervention, to reduce plasma lipids (cholesterol, LDL-C, TG), in the context of growing concern about hyperlipidemia and hypercholesterolemia and their consequences [1]. The quantity of favorable experimental evidence, supported by epidemiological and clinical studies, resulted in the approval of a health claim by the FDA in 1999 [2], which suggested the inclusion of 25 grams of soy protein in the daily diet to reduce cardiovascular disease. More recent data confirm this and link the consumption of soy protein with a lower incidence of chronic diseases [3].

The main constituent of the soy proteins are the globulins, divided into 2 types by their sedimentation coefficients: 7S or beta-conglycinin and 11S or glycinin. They constitute approximately 90% of the total protein of the seed. The whole protein isolate and β-conglycinin (7S protein) fraction have received much greater attention in scientific research than other protein fractions [3-6], reserving a promising field of research for glycinin, despite its being a major fraction of the seed.

Studies with isolated soy protein in hypercholesterolemic rats indicate that, in addition to lowering serum cholesterol, it decreases serum triglycerides levels. One possible mechanism for the cholesterol lowering effect was raised by Lovati et al. [7,8], indicating that soy protein could modulate the levels of hepatic LDL-C receptors, suppressed in hypercholesterolemia.

On the other hand, the advent of drugs with an inhibitory effect on 3-hydroxy-3 methylglutaryl-CoA (HMG-CoA) reductase, known as statins, to treat lipoprotein metabolism disorders, is a significant event in the history of prevention therapy, specifically in the prevention of atherosclerosis, with consistent benefits with regard to cardiovascular disease mortality [9]. In addition to reducing LDL cholesterol and raising HDL-C levels, statins suppress inflammation, in particular reducing C-reactive protein, a biological marker which, when elevated, indicates a risk of heart attack [10]. Among the statins, rosuvastatin has shown a greater reduction of LDL-C than other statins in clinical trials, and helps to slow the progress of atherosclerosis, reducing the formation of new lesions and the incidence of coronary events [9,10].

In the study of hypercholesterolemia, it is known that compounds present in foods, which are designated functional compounds, can assist the drug to reduce and/or prevent many metabolic disorders related to increased lipids in the circulation. However, these compounds can have a non-synergistic effect that affects the availability of the drug and thus impairs therapy or reduces the effect of functional compounds.

In order to collect evidence on this complex mechanism, we investigated the effects of ingesting a daily dose of isolated glycinin soy protein (11S protein), alone or combined with rosuvastatin (a statin drug), on the control of hypercholesterolemia in experimental animals.

## Methods

### Chemical composition of soy flour

The chemical composition of defatted soy flour was determined by AOAC methods (1998) [11].

### Isolation of 11S globulin

Commercial soy flour (grain size 60 mesh) was defatted with hexane (ratio 1:8 w/v), stirred for a period of 4 hours at room temperature. This procedure was repeated (ratio 1:4 w/v). Subsequently, the flour was filtered and dried at room temperature for 24 hours. The 11S globulin was isolated by the procedure reported in Nagano et al. [12], adapted by Ferreira et al. [6]. The protein content was determined by the method of Lowry et al. [13], with bovine serum albumin as standard protein (Sigma Chemical Co., St. Louis, MO, USA). The number and the molecular mass of the 11S protein subunits were estimated by electrophoresis in 10% polyacrylamide gel with 1% sodium dodecyl sulfate, in a discontinuous pH system, as described by Laemmli [14], in the presence of the reducing agent 2-mercaptoethanol. The standard protein mixture of known molecular weights contained phosphorylase b (97 kDa), bovine serum albumin (66 kDa), ovalbumin (45 kDa), carbonic anhydrase (30 kDa), soybean trypsin inhibitor (21.5 kDa) and lactalbumin (14.4 kDa).

### Biological evaluation

Forty-five male Wistar rats (*Rattus norvegicus *var. *albinus*) from the Central Animal House at São Paulo State University (UNESP) at Botucatu (SP, Brazil) were kept in a controlled environment (12:12 h photoperiod, temperature 23 ± 2°C and relative humidity 60 ± 5%) in individual metabolic cages, with free access to standard Purina^® ^(São Paulo, SP, Brazil) chow and water, for approximately 14 days, to adapt to surroundings and reach an average weight of 180 to 200 grams. They were then separated into five dietary groups (*n *= 9) (Table 1): 1) standard group (STD), given a diet containing casein as protein source, as recommended by the American Institute of Nutrition (AIN-93M) [15]; 2) hypercholesterolemic (HC), given the standard diet plus 1% cholesterol and 0.5% cholic acid, as described by Ferreira et al. [6]; 3) HC+11S, given the hypercholesterolemic diet plus 11S soy protein (300 mg/kg/day); 4) HC+ROS, given the HC diet and the drug rosuvastatin (10 mg/kg/day); 5) HC+11S+ROS, given the HC diet, the 11S protein and the drug. The experimental period was 28 days. The glycinin soy protein and drug were dissolved in saline and administered by gavage daily at different periods. The procedures were performed in accordance with the Guide for the Care and Use of Laboratory Animals [16]. The experimental procedure was approved by the Research Ethics Committee (Res. No. 32/2008).

**Table 1:** Compositions of experimental diets

Ingredients	STD	HC	HC+11S	HC+ROS	HC+11S+ ROS
Casein^1^	14.74	14.74	14.74	14.74	14.74
Starch	45.83	44.33	44.33	44.33	44.33
Starch dextrinated	15.5	15.5	15.5	15.5	15.5
Sucrose	10	10	10	10	10
Soybean oil	4	4	4	4	4
Cellulose	5	5	5	5	5
Mineral mix^2^	3.5	3.5	3.5	3.5	3.5
Vitamin mixture^2^	1	1	1	1	1
L-Cystine	0.18	0.18	0.18	0.18	0.18
Choline bitartrate	0.25	0.25	0.25	0.25	0.25
Cholesterol^3^	0	1	1	1	1
Cholic acid^1^	0	0.5	0.5	0.5	0.5
*Treatment*					
Glycinin (mg/kg/dia)	-	-	300	-	300
Rosuvastatin (mg/kg/dia)	-	-	-	10	10

STD = standard diet, AIN-93 Composition; HC = hypercholesterolemic diet: AIN-93 plus 1 g/100 g cholesterol and 0.5 g/100 g cholic acid; HC+11S = HC diet + glycinin (300 mg/kg/day); HC+ROS = HC diet + rosuvastatin (10 mg/kg/day); HC+11S+ROS = HC diet + glycinin (300 mg/kg/day) and rosuvastatin (10 mg/kg/day). ^1^Sigma-Aldrich, Co., USA. ^2^PragSoluções^®^, Co., Brazil. ^3^Reagen, Co., USA.

The variations in body weight and food intake were checked daily during the 28-day period. At the end of the experiment and after 12 hours of fasting, animals were sacrificed by decapitation. Blood was collected into tubes containing SST gel separator II (BD Vacutainer^® ^B, Franklin Lakes, NJ, USA) and then centrifuged at 1, 900 × *g *for 15 minutes at 4°C. Serum was collected and stored at -20°C for subsequent biochemical analysis. The liver was removed, washed in saline (9 g NaCl/L), weighed and immediately stored frozen at -20°C. The hepatosomatic index (HSI) was calculated as (liver weight/body weight) × 100.

### Analysis of plasma and liver

Total cholesterol (TC), HDL-cholesterol (HDL-C) and triglyceride (TG) levels were analyzed in triplicate with Labtest^® ^(Laborlab^®^. São Paulo. SP, Brazil) enzymatic kits. The fraction of non-HDL-C (LDL-C + VLDL-C) was calculated from the difference between total cholesterol and HDL-C and the atherogenic index was calculated as (TC - HDL-C)/HDL-C.

The TC and TG levels in the liver were determined as described by Haug and Hostmark [17] and the liver biochemical tests were performed as in the plasma analysis. The hepatosomatic index (HSI) was calculated as (liver weight/body weight × 100).

### Statistical analysis

Data were analyzed with the program SigmaStat^® ^3.5 (Dundas Software, Erkrath, Germany, 1999), using analysis of variance (ANOVA) and the Student-Newman-Keuls (SNK) test for multiple comparison between treatments. Significance was accepted when p < 0.05. All results were presented as mean ± standard error (SEM).

## Results

The protein content of the defatted soy flour used to extract the glycinin was 51%. According to Wolf et al. [18], the minimum amount of protein in soy flour ranges from 40 to 50%, depending on the amount of fat and carbohydrate and also the conditions of cultivation. The protein, treated with reducing agent during electrophoresis, was resolved into bands with molecular weights ranging from 22.07 to 58.14 kDa, as reported by Ferreira et al. [6]. According to Nagano et al. [12], the purity of the 11S globulin fraction obtained by this method is greater than 90%.

Hypercholesterolemia was induced in adult rats by adding 1% cholesterol and 0.5% cholic acid to the standard diet AIN-93M, as proposed in [7,19-22], and this was an effective model of hypercholesterolemia in the groups HC, HC+11S, HC+ROS and HC+11S+ROS, as indicated by the results described below.

After 28 days of the experiment, there was no statistical difference (p < 0.05) in weight gain among animals, although there were differences in food consumption between all the hypercholesterolemic groups and group STD, as shown in Table 2. Thus, we can infer that the supply of glycinin by gavage (300 mg/kg/day), although it represented an addition of around 2.8% of dietary protein intake, did not lead to weight change and may be considered a supplement that could influence lipid metabolism.

**Table 2:** Body weight and food intake in experimental groups

	STD	HC	HC+11S	HC+ROS	HC+11S+ROS
**Initial weight (g)**	195.23 ± 5.03ª	196.55 ± 4.63ª	194.55 ± 3.92ª	193.6 ± 2.31ª	193.44 ± 2.35ª
**Final weight (g)**	287.77 ± 7.07ª	295.01 ± 5.43ª	298.53 ± 5.26ª	306.5 ± 3.79ª	301.49 ± 3.88ª
**Weight gain (g/day)**	3.31 ± 0.24ª	3.51 ± 0.32ª	3.71 ± 0.22ª	4.02 ± 0.11ª	3.84 ± 0.10ª
**Food consumption (g/day)**	15.47 ± 0.27^b^	16.74 ± 0.33ª	17.27 ± 0.29^a^	18.86 ± 0.14^a^	18.34 ± 0.18^a^

STD = standard diet, AIN-93 Composition; HC = hypercholesterolemic diet: AIN-93 plus 1 g/100 g cholesterol and 0.5 g/100 g cholic acid; HC+11S = HC diet + glycinin (300 mg/kg/day); HC+ROS = HC diet + rosuvastatin (10 mg/kg/day); HC+11S+ROS = HC diet + glycinin (300 mg/kg/day) and rosuvastatin (10 mg/kg/day). Values represented as mean ± SEM. Different superscript letters in same row indicate a significant difference (p < 0.05) between experimental groups.

Table 3 indicates a significant increase (171.8%) in total plasma cholesterol in the HC group, relative to the standard group, STD, showing that increased levels of cholesterol in the diet contribute significantly to the elevation of plasma cholesterol. Group HC+11S showed a reduction in total cholesterol of 11.1%, compared to HC, while group HC+ROS showed a reduction of 25.9%. These results suggest that oral administration of glycinin, although less powerfully than the drug at these doses, lowered the cholesterol appreciably, since it is recognized that a 10% reduction in serum cholesterol translates to a 15% reduction in risk of mortality from coronary heart disease [9]. The condition of hypercholesterolemia was evident in the increased non-HDL fraction in the plasma of the HC group, while HDL-C remained unchanged, relative to STD. These changes resemble the typical situation of hyperlipidemia in humans, which may be associated with a reduction in the number of LDL-C receptors.

**Table 3:** Effect of diets on plasma lipid profile and hepatic lipid content in rats

	STD	HC	HC+11S	HC+ROS	HC+11S+ ROS
**Plasma**					
**TC (mmol/L)**	1.56 ± 0.07^b^	4.24 ± 0.45ª	3.77 ± 0.41ª	3.14 ± 0.12ª	4.33 ± 0.40ª
**HDL-C (mmol/L)**	0.78 ± 0.03^b^	0.82 ± 0.06^b^	1.04 ± 0.04ª	0.73 ± 0.04^b^	0.73 ± 0.08^b^
**non-HDL-C (mmol/L)**	0.78 ± 0.04^b^	3.42 ± 0.47ª	2.74 ± 0.4ª	2.43 ± 0.16ª	3.57 ± 0.42ª
**TG (mmol/L)**	0.48 ± 0.02^b^	1.09 ± 0.12ª	0.85 ± 0.11ª	0.84 ± 0.07ª	0.81 ± 0.08ª
**Liver**					
**TC (μmol/g)**	7.15 ± 0.34^d^	57.66 ± 1.06^b^	54.97 ± 2.12^b^	36.15 ± 2.12^c^	76.66 ± 2.12ª
**TG (μmol/g)**	14.52 ± 0.91^c^	58.77 ± 3.97ª	46.48 ± 4.06^b^	41.35 ± 2.43^b^	46.74 ± 3.08^b^
**Atherogenic index**	1.01 ± 0.05^c^	4.62 ± 0.87^ab^	2.67 ± 0.38^bc^	3.49 ± 0.42^ab^	5.06 ± 0.81^a^

STD = standard diet, AIN-93 Composition; HC = hypercholesterolemic diet: AIN-93 plus 1 g/100 g cholesterol and 0.5 g/100 g cholic acid; HC+11S = HC diet + glycinin (300 mg/kg/day); HC+ROS = HC diet + rosuvastatin (10 mg/kg/day); HC+11S+ROS = HC diet + glycinin (300 mg/kg/day) and rosuvastatin (10 mg/kg/day). Values are represented as mean ± SEM. TC = total cholesterol; HDL-C = high density lipoprotein; non-HDL-C = difference between TC and HDL-C; TG = triglycerides; atherogenic index = non-HDL-C/HDL-C. Different superscript letters in the same row indicate a significant difference (p < 0.05) between experimental groups.

The HDL-C, however, showed an increase of 26.8% in group HC+11S, relative both to the standard group STD, and to HC. This result was significant, since the groups HC+ROS and HC+11S+ROS showed no difference from the HC group, and supports the hypothesis that glycinin acts by raising HDL-C, which, by carrying cholesterol esters from peripheral tissues to the liver, would be a protective factor in reducing the risk of cardiac events.

HC group showed an increase of 127.1% in plasma TG, compared to the level in the STD group. The other groups (HC+11S, HC+ROS and HC+11S+ROS) showed a reduction of approximately 23% of the level in the HC group.

There was a significant increase (338.5%) of the non-HDL fraction in HC, relative to the standard group STD, while the levels in groups HC+11S and HC+ROS were reduced by 19.88% and 28.95%, respectively, from that in HC. However, group HC+11S+ROS did not differ from the HC group, suggesting that the actions of the isolated protein and the drug were not additives when they were administered in combination.

In this study, the atherogenic index, a possible indicator of a predisposition to heart disease, was 357.4% higher in the HC group than in the standard group, STD. Marked decreases of 42.2% in group HC+11S and 24.5% in HC+ROS, compared to HC, were observed in this index, but the same response was not observed in HC+11S+ROS, which showed, surprisingly, a greater atherogenic index than group HC.

An increase ranging from 24% to 30% was observed in the liver weight of groups HC, HC+11S, HC+ROS and HC+11+ROS, compared to the standard casein group, STD (Table 4). However, there was no significant difference among the groups HC+11S, HC+ROS and HC+11S+ROS. Thus, both the liver weight and the hepatosomatic index are inconclusive parameters for this analysis, as evidenced by various results described in the literature [7,20,21,23].

**Table 4:** The liver weight and hepatosomatic index (HSI) of experimental groups

	STD	HC	HC+11S	HC+ROS	HC+11S+ROS
**Liver (g)**	10.90 ± 0.45^c^	13.54 ± 0.36^b^	17.63 ± 0.28ª	17.07 ± 0.92ª	17.14 ± 0.78ª
**HSI (wt %)**	3.79 ± 0.12^c^	4.59 ± 0.14^b^	5.91 ± 0.12ª	5.56 ± 0.27ª	5.68 ± 0.22ª

HSI = (liver weight/body weight × 100); STD = standard diet, AIN-93 Composition; HC = hypercholesterolemic diet: AIN-93 plus 1 g/100 g cholesterol and 0.5 g/100 g cholic acid; HC+11S = HC diet + glycinin (300 mg/kg/day); HC+ROS = HC diet + rosuvastatin (10 mg/kg/day); HC+11S+ROS = HC diet + glycinin (300 mg/kg/day) and rosuvastatin (10 mg/kg/day). Values represented as mean ± SEM. Different superscript letters in the same row indicate a significant difference (p < 0.05) between experimental groups.

The animals that received the hypercholesterolemic diet (HC, HC+11S, HC+11S+ ROS) presented higher levels of total cholesterol (TC) and triglycerides (TG) in the liver than those given a standard diet (STD), as indicated in Table 3. However, the reduction of serum cholesterol observed in the HC+11S group, relative to the HC group, was not reflected in the liver, as also observed by Lovati et al. [7]. In contrast, a reduction of hepatic cholesterol on the order of 37.3% was observed in the HC+ROS group, relative to HC. Conversely, group HC+11S+ROS showed a significant increase in TC relative to HC, which denotes an interference or inhibition of the effects of the drug by the presence of 11S soy protein, when both were administered together.

Hepatic TG showed a very significant increase (304.8%) in group HC, relative to the STD group, while the other groups (HC+11S, HC+ROS and HC+11S+ROS) showed significant reductions of 20.9%, 29.7% and 20.5%, respectively, relative to HC.

## Discussion

A large body of evidence indicates that the type of protein in the diet, particularly those found in the legumes, can affect plasma cholesterol levels in animal models and humans [1,6,19,23-25]. Isolated soy protein has given outstandingly positive results, particularly in studies of hypercholesterolemic rats, where, in addition to lowering serum cholesterol, it was observed to reduce TG [20,21]. With regard to the isolated protein fractions, the 7S globulin has been studied the most [3-6,19,26,27]. Thus, we aimed in this experiment to show the role of 11S globulin in this context, which, in a single daily dose, resulted in a reduction of atherogenic factors, including TC and TG. This corroborates a published study [7] on the effect of soy globulins *in vitro *and *in vivo*, which found that Sprague-Dawley rats fed on a hypercholesterolemic diet, with casein as the protein source, showed a reduction in plasma cholesterol of 34.84% and 32.70% after administration by gavage of 7S and 11S globulin, respectively, at a dose of 30 mg soy protein per day. Moreover, Adams et al. [28] observed a 41% increase in HDL-C when isolated soy protein was introduced as a protein source in place of casein and lactalbumin in the diet of monkeys, but there was no improvement in lipoprotein levels or cardiovascular biomarkers when the soy protein was replaced by 7S or 11S globulin.

The reduction in plasma triglyceride levels in the animals receiving the glycinin was similar to that reported by Aoyama et al. [29] in Sprague-Dawley rats fed isolated soy protein or its hydrolyzates, which showed 24.81% and 33.33%, respectively, lower plasma triglycerides than the casein control group. Fukui et al. [20,21] also showed reduced levels of plasma triglycerides in Sprague-Dawley rats fed a diet enriched with cholesterol and treated with isolated soy protein, compared with the group that received casein, the reduction varying between 18.18% and 37.65%. It was suggested by Kingman et al. [30] that the reduction in TG occurs independently of the level of cholesterol.

The most significant result of the experiment was the increase in plasma HDL-C in the HC+11S group, suggesting that the 11S protein fraction may be involved in regulatory processes: Perret et al. [31] mentions an inverse relationship between the cholesterol content of hepatocytes, mRNA and HDL-C levels. Rho et al. [32] noted increased plasma concentrations of HDL-C in Sprague-Dawley rats treated with peptides derived from black soy, in various concentrations, relative to a casein diet. The significant rise in HDL-C in the HC+11S group was not observed in the HC+ROS group, since statins are effective in lowering LDL-C, but have only a modest effect in raising levels of HDL-C [10]. The results of the HC+11S group suggest a protective role for 11S globulin against the potential damage caused by excess cholesterol in the body, since circulating HDL-C has an antiatherogenic effect, in contrast to LDL-C, which is a fraction considered to be an important cardiovascular risk factor.

Among the groups studied, only in HC+11S the atherogenic index did not differ from the STD group. In biological terms, this is a significant evidence that 11S globulin is a protective factor, improving the atherogenic index in animals exposed to a hypercholesterolemic diet, since a low LDL/HDL ratio and low levels of plasma triglycerides are known to decrease appreciably the risk of cardiovascular disease [33]. Rho et al. [32] observed a reduction in the atherogenic index in Sprague-Dawley rats treated with various concentrations of peptides derived from soy, relative to the casein control group. Similarly, in a human study, there was a lower LDL-C/HDL-C ratio in individuals with moderate hypercholesterolemia who received isolated soy protein than in a group consuming animal protein [34].

The reduction of the non-HDL-C fraction demonstrated in the HC+ROS group is similar to literature values for LDL-C in subjects taking the usual dose (5 to 10 mg) of rosuvastatin, which reduces LDL-C by 30 to 40% [9]. Also, we observed a significant reduction of hepatic cholesterol in this group. However, this reduction was completely inhibited, and the non-HDL-C level exceeded that of HC group, when the 11S was given concomitantly with rosuvastatin (HC+11S+ROS). Thus, the results of this study suggest a possible antagonistic effect between the drug and the protein, as noted in the levels of plasma total cholesterol, in non-HDL cholesterol fraction, and the significant increase of cholesterol in the liver, and that the separate administration of glycinin and rosuvastatin by different routes did not prevent this antagonism.

In relation to hepatic triglycerides, the groups HC+11S, HC+ROS and HC+11S+ROS all showed lower levels than the HC group. Previous studies [32,35] also showed a reduction of hepatic triglycerides in rats treated with soy protein, compared to rats fed on casein as protein source, suggesting that this effect could result from the regulation of SREBP-1c and its target genes involved in fatty acid synthesis.

Possible mechanisms for the cholesterol-lowering effect have been proposed [7,8], suggesting that soy protein could modulate the levels of LDL-C receptors, while others [22,26,36], attribute this role to the peptides formed during digestion of the protein. Such findings are supported in clinical trials that ascribe reduce LDL-C to the administration of peptides derived from soy protein [37].

Significant progress has been recorded in the identification of a specific mechanism leading to the reduction of lipid concentrations by statins, particularly rosuvastatin, namely the observed inhibition of 3-hydroxy-3-methyl glutaryl CoA reductase, the rate-limiting step in the synthesis of cholesterol, which significantly reduces LDL-C [9].

Moreover, soy protein, in the form of isolated soy protein, or given as separate 7S and 11S proteins, has shown satisfactory results, particularly in lowering total cholesterol and increasing HDL-C, although its mechanisms of action are still inconclusive. However, there is still no significant synergistic effect, when drugs and functional food are combined, that could be used to limit the dosage of the drug, reducing the possible side effects and costs associated with managing chronic diseases, among other benefits.

In this study, when the effects of the isolated 11S protein (HC+11S group), the drug alone (HC+ROS) and the combined treatment (HC+11S+ROS) were compared, the beneficial effects of the separate treatments were observed to disappear in the combination, showing that there is no synergy between them.

In conclusion, the present results suggest the participation of glycinin in raising the plasma HDL-C and significantly decreasing a number of pro-atherogenic factors in rats that received the hypercholesterolemic diet, indicating that it may be considered a functional compound, capable of improving the profile of plasma lipoproteins, which may have an important role to play in reducing the risk of heart disease. However, a possible drug-glycinin interference shows that further investigation into the mechanism of possible interactions between drugs and food proteins is necessary, to avoid inhibition of the therapeutic benefits and possible adverse effects. These findings demonstrate the need for further research to clarify the issue, since soy products are customary in the current menu and could alter the pharmacological effect of any treatment. Studies are in progress to identify the effects of peptides derived from 11S and their role in cholesterol metabolism.

## Competing interests

The authors declare that they have no competing interests.

## Authors' contributions

PGF carried out the animal care, all experiments and analysis, data processing and wrote the manuscript. RWN participated in the protein extraction, animal care and the measurement of food intake and animal sacrifice. ESF assisted in the protein extraction. MAS participated in the biochemical analyses and animal sacrifice. VAN conceived of the study and its design and helped to draft the manuscript. AD conceived of the study, data analyses and design and wrote the manuscript. All authors read and approved the final version of the manuscript.
